# Prediction of attempted suicide in men and women with crack-cocaine use disorder in Brazil

**DOI:** 10.1371/journal.pone.0232242

**Published:** 2020-05-04

**Authors:** Vinícius Serafini Roglio, Eduardo Nunes Borges, Francisco Diego Rabelo-da-Ponte, Felipe Ornell, Juliana Nichterwitz Scherer, Jaqueline Bohrer Schuch, Ives Cavalcante Passos, Breno Sanvicente-Vieira, Rodrigo Grassi-Oliveira, Lisia von Diemen, Flavio Pechansky, Felix Henrique Paim Kessler

**Affiliations:** 1 Center for Drug and Alcohol Research, Hospital de Clínicas de Porto Alegre, Universidade Federal do Rio Grande do Sul, Porto Alegre, Brazil; 2 Graduate Program in Psychiatry and Behavioral Sciences, Universidade Federal do Rio Grande do Sul, Porto Alegre, RS, Brazil; 3 Center for Computational Sciences, Universidade Federal do Rio Grande, Porto Alegre, Brazil; 4 Molecular Psychiatry Laboratory, Hospital de Clínicas de Porto Alegre, Universidade Federal do Rio Grande do Sul, Porto Alegre, Brazil; 5 Developmental Cognitive Neuroscience Lab, Pontifical Catholic University of Rio Grande do Sul, Porto Alegre, Brazil; University of Toronto, CANADA

## Abstract

**Background:**

Suicide is a severe health problem, with high rates in individuals with addiction. Considering the lack of studies exploring suicide predictors in this population, we aimed to investigate factors associated with attempted suicide in inpatients diagnosed with cocaine use disorder using two analytical approaches.

**Methods:**

This is a cross-sectional study using a secondary database with 247 men and 442 women hospitalized for cocaine use disorder. Clinical assessment included the Addiction Severity Index, the Childhood Trauma Questionnaire, and the Structured Clinical Interview for the Diagnostic and Statistical Manual of Mental Disorders, totalling 58 variables. Descriptive Poisson regression and predictive Random Forest algorithm were used complementarily to estimate prevalence ratios and to build prediction models, respectively. All analyses were stratified by gender.

**Results:**

The prevalence of attempted suicide was 34% for men and 50% for women. In both genders, depression (PR_M_ = 1.56, PR_W_ = 1.27) and hallucinations (PR_M_ = 1.80, PR_W_ = 1.39) were factors associated with attempted suicide. Other specific factors were found for men and women, such as childhood trauma, aggression, and drug use severity. The men's predictive model had prediction statistics of AUC = 0.68, Acc. = 0.66, Sens. = 0.82, Spec. = 0.50, PPV = 0.47 and NPV = 0.84. This model identified several variables as important predictors, mainly related to drug use severity. The women's model had higher predictive power (AUC = 0.73 and all other statistics were equal to 0.71) and was parsimonious.

**Conclusions:**

Our findings indicate that attempted suicide is associated with depression, hallucinations and childhood trauma in both genders. Also, it suggests that severity of drug use may be a moderator between predictors and suicide among men, while psychiatric issues shown to be more important for women.

## Introduction

Suicide is a complex global health problem that currently accounts for 1.4% of premature deaths worldwide. According to the World Health Organization, a total of 800,000 suicides are documented each year, which corresponds to one completed suicide every 40 seconds. Unlike many diseases and risk behaviors, the mortality rate has not declined over the past two decades [1]. For instance, suicide is among the top three causes of death of people aged 15–44 [2]. Suicide prevalence rates differ by country, varying according to the heterogeneity of cultural, social, economic, and environmental factors [3]. Furthermore, each of the components of suicidal behavior, such as ideation, attempt, and mortality, also have different epidemiology [4]. The etiology of suicide is heterogeneous, although the literature shows that previous suicide attempts and the presence of psychiatric disorders are the most established predictors of suicide [5–8]. Within this category, substance use disorders (SUD), such as cocaine and alcohol use disorders, are among the most significant predictors of suicide [9].

In Brazil, crack-cocaine use and suicidal behavior are public health problems [10–12]. A recently large-scale study with a representative sample of Brazilians found rates of suicidal attempts and deaths in the general population of 9.9 and 5.4%, respectively. In crack-cocaine users these rates were significantly higher, 40.0 and 20.8%, respectively [13]. Previous studies have shown that 47% of crack-cocaine users had a current suicide risk [14], and a prevalence of suicidal behaviors of 30% in crack-cocaine addicts, in Brazil. The subject of suicide has been the focus of studies in psychiatry in the last decades, but the understanding about this behavior remains insufficient [15]. Moreover, the predictive factors in this vulnerable population were not well explored yet. These individuals present multiple psychosocial vulnerabilities, high rates of clinical and psychiatric comorbidities, and low adherence to treatment [16–19], which may have a significant impact on suicidal behavior. Furthermore, crack and cocaine are the illicit drugs that most lead to demand for detoxification treatment in psychiatric facilities [20,21], generating considerable cost to the public health system.

Gender-related aspects also seem to influence or mediate the drug use profile and the severity of crack-cocaine dependence [22,23] and may be potentially associated with suicide [3,24–26]. Although some predictors of suicide are common to both genders, such as prior mental disorders, substance abuse, and early exposure to violence, some other potential risk factors appear to be gender-specific [26]. Female-specific risk factors are related to physical and sexual abuse, eating disorders, bipolar disorder, depressive symptoms, interpersonal problems and previous abortion. Male-specific risk factors are often related to disruptive behavior, hopelessness, family or friend history of suicide, mental disorders due to alcohol and drug abuse, externalizing disorders, homelessness, and access to means [27,28]. Considering the differences in suicidal behavior between men and women, targeted and tailored prevention and intervention strategies are recommended [24]. However, only a few studies have evaluated gender differences related to suicide, especially among cocaine users, which is still a gap to be explored in the field of psychiatry.

Traditional statistical methods have provided valuable insights into addiction psychiatry. However, few papers have explored the complex relationship between suicide and its predictors in men and women with cocaine use disorder [29]. The use of different analytical methods, such as machine learning, to complement each other, may be an essential step towards better understanding the phenomenon of suicide, bringing advances to the field [30] and providing a foundation for changes to treatment protocols.

This study is driven by the hypothesis that the characteristics of subjects who attempt suicide differ between men and women with cocaine use disorder, who have distinct psychiatric and substance use profiles. We aim to investigate associated factors and predictors of suicide attempts in cocaine use disorder inpatients, through descriptive and predictive models using secondary databases obtained from retrospective cross-sectional studies, and stratifying by gender.

## Methods

Analytical procedures were organized in distinct phases according to the Knowledge Discovery in Databases (KDD) methodology [31]. First, we presented information about study participants and data selection. Then, we detailed preprocessing and data transformation, including creation of new variables. Finally, we presented all statistical analyses and data mining, including configuration of the machine learning algorithms, solutions to the problem of class imbalance, evaluation measures, and the software packages used in the implementation.

### Participants and assessments

Our data sources are secondary databases populated with data from 2012 to 2018 from two inpatient units specialized in addiction treatment in Porto Alegre, Southern Brazil. This study was approved by the Hospital de Clínicas de Porto Alegre (HCPA) Research Ethics Committee (number 180166). The inclusion criteria were as follows: (1) fulfilled DSM-IV criteria for cocaine use disorder; (2) reporting crack-cocaine as the preferred drug in case of multiple drug use; (3) 18 or more years of age; (4) absence of cognitive deficits that impair the reliability of the answers; and (5) voluntarily acceptance of all research procedures via written informed consent.

Some clinical variables were extracted from the hospital medical records, but most data were collected using three instruments: the sixth version of the Addiction Severity Index (ASI-6) [32,33]; the Structured Clinical Interview for DSM-IV axis I disorders (SCID-I) [34]; and the Childhood Trauma Questionnaire (CTQ) [35,36]. Trained researchers assessed all participants by administration of the Brazilian versions of clinical and semi-structured interviews. Details of the data collection procedure are described elsewhere [37,38]. The initial sample comprised 863 individuals who met the inclusion criteria. We pre-selected a set of variables related to suicide according to recent literature reviews [39,40].

### Preprocessing and data transformation

The data sample selected in the previous phase was preprocessed in order to clean and standardize variable types, formats, and content. We then created new variables based on transformations and combinations of the original ones. Supplementary material S1 Text presents detailed information on the method for creating these variables. We included sociodemographic variables; tuberculosis, hepatitis and HIV infection, and number of other chronic diseases; life proportion since first treatment for alcohol or drugs; years and life proportion of regular use of alcohol, cannabis, cocaine (snorted and smoked), and tobacco; age at first use of each substance; number of distinct psychoactive substances used for more than 50 days during lifetime; alcohol and drug craving and withdrawal symptoms at the time of hospitalization; social support comprising partner, adult relatives and/or close friends; close relationship(s) with other drug user(s); difficulty in controlling aggressiveness, talking about feelings, and enjoying leisure time; physical abuse (childhood and lifetime); sexual abuse (childhood and lifetime); dichotomized CTQ scores; idealization of upbringing, based on the statements that are not used to compute trauma scores in the CTQ; SCID-I diagnoses grouped and dichotomized as psychotic, bipolar, depression, obsessive-compulsive, post-traumatic stress, alcohol use, eating and anxiety disorders. The outcome of attempted suicide is binary with answers: “no suicide attempts in lifetime” and “at least one suicide attempt (under drug effect or not).”

We also removed noisy instances and individuals with sparse information. We excluded 42 subjects because the outcome was missing. Several other participants were removed because the instruments were not sufficiently complete: 38 for the ASI-6, 80 for the SCID-I, and 34 for the CTQ. We then dropped all variables with more than 7% missing values. This threshold was chosen based on analysis of the missing data pattern presented in S1 Fig. At this point, we observed that only 4 out of 57 variables had more than 3% of data missing. The final sample comprised 57 variables on 669 inpatients (247 men and 422 women), representing our study population.

### Data mining and statistical analysis

In this study, we adopted two distinct approaches to analyze data. We first performed a Generalized Linear Model (GLM) with Poisson distribution, log link, and robust estimation of variance to calculate attempted suicide prevalence ratios (PR), to investigate risk factors. PR was chosen as the ideal statistic rather than the odds ratio because it is considered more adequate for our study design, and interpretation is more intuitive [41]. With this approach, we aimed to descriptively explore variables associated with attempted suicide using a partitioned deviance statistical method. This analysis was performed with the whole sample (still stratifying by gender). Associations between suicide attempt and all variables were investigated through PR estimations controlled for age and ethnicity. Then, all statistically significant variables, along with age, were used as independent variables in a descriptive multiple GLM. Finally, stepwise selection with backward elimination was applied until all main effect p-values were < 0.2 (except for age, used to control).

#### Machine learning approach

The second approach was a supervised machine learning algorithm called Random Forest (a type of predictive modelling), which requires a data preparation. The dataset was randomly split into distinct partitions, 80% for training/validating and 20% for testing. We ran the Multivariate Imputation by Chained Equations (MICE) algorithm [42] to impute missing training data, setting the number of iterations equal to 10, and used the Random Forest imputations method for all variables. A detailed description of data imputation is presented in the supplementary material and illustrated in S2 Fig.

We applied the Recursive Feature Elimination (RFE) algorithm [43] to reduce the complexity of the predictive model, by retaining only the variables that most predict attempted suicide. This algorithm starts by fitting the model to all predictors. At each iteration, the features are ranked using their importance to prediction. The least relevant feature is removed, and the model is refitted. The set of features with the best performance is determined, and they are used to learn the final model.

Random Forest [44] is an ensemble method for supervised learning. It grows hundreds of different decision trees and combines them as a single classifier using majority voting. Each tree grows using a bootstrap of the training set and random search of best splits, parameterized by the number of candidate predictors. We used repeated cross-validation [45] for all learned models. In this schema, the original training dataset is randomly partitioned into *k* equal size samples, where each one is used as the validation data, with the remaining samples used as training data. This procedure is repeated *n* times yielding several random partitions of the original training dataset. The results are averaged to produce a single prediction quality estimation. The advantage of this method is that all examples are used for both training and validation in several distinct models. We used *k* = 10 and *n* = 5 as resampling method for RFE and for tuning the Random Forest hyperparameter. We varied the randomly selected predictors using a grid search incrementing its value from 1 to $1.5\sqrt{sf}$, where *sf* is the number of selected features. We used the area under the Receiver Operating Characteristic (ROC) curve (AUC) [46] to evaluate prediction quality. The number of candidate predictors with the best AUC was selected to fit the model with all training data.

#### Class imbalance problem

When the frequency of suicide attempts had a significant negative impact on model fitting, we dealt with the class imbalance problem by subsampling the training data using three distinct strategies: down-sampling that randomly samples the majority class to be the same size as the minority; up-sampling that randomly samples with replacement the minority class to be the same size as the majority; and SMOTE [47], which is a hybrid technique that down-samples the majority class and synthesizes new data points in the minority.

#### Assessment of model performance

Final predictive models were evaluated using the following quality measures: AUC, Balanced Accuracy, Sensitivity, Specificity, Positive Predictive Value (PPV), and Negative Predictive Value (NPV). The classification threshold was defined as the point that maximized the sum of Sensitivity and Specificity. Additionally, the result of a statistical test of difference of proportions upon the null hypothesis of equality between No Information Rate (NIR) and Accuracy was reported. NIR is the best guess given no information beyond the overall distribution of the outcome, i.e., it is the prevalence of the majority category.

### Implementation

Data curation and GLM were implemented using IBM SPSS syntax because the original databases were stored in SPSS native format. All the procedures and analyses of remaining phases of the KDD process were implemented in R scripts using RStudio IDE and the packages VIM (visualize missing data), mice (imputation), DMwR (SMOTE sampling), caret (training), randomForest (predictive algorithm), and pROC (plot ROC curves).

We decided to stratify all analyses by gender because suicide is a phenomenon with signatures that differ between genders in terms of the lethality of the method used, psychosocial risk factors and prevalence of psychiatric disorders [24,48].

## Results

### Descriptive approach

Table 1 presents the sample's sociodemographic profile and the prevalence rates of attempted suicide within those variables, stratified by gender and controlled by age and ethnicity. The overall prevalence of attempted suicide was 44%, 34% for men and 50% for women. For men, there was a significant association between age and suicide attempt, showing a 3% increase in prevalence of attempted suicide for each incremental year of age. No other direct relationship was found between the outcome and any other sociodemographic variables.

**Table 1:** Sociodemographic variables and prevalence ratios (PR) for attempted suicide, stratified by gender.

	Gender*		Attempted suicide**					
	Men	Women	Men	PR	p	Women	PR	P
**Age (years)** ^**1**^	34.3 ± 8.5	31.4 ± 8.7	-	1.02	0.03	-	1.00	0.551
**Ethnicity** ^**2**^								
Black	50 (20.5)	172 (40.8)	19 (38.0)	ref.	-	77 (44.8)	ref.	-
Multiracial Brazilians	54 (22.1)	98 (23.2)	14 (25.9)	0.72	0.252	53 (54.1)	1.20	0.158
White Latin American	140 (57.4)	152 (36.0)	52 (37.1)	0.95	0.812	81 (53.3)	1.18	0.153
**Education** ^**2**^								
None	58 (23.6)	147 (35.9)	20 (34.5)	ref.	-	77 (52.4)	ref.	-
Basic Education	109 (44.3)	153 (37.4)	39 (35.8)	1.15	0.523	66 (43.1)	0.82	0.111
Secondary Education	79 (32.1)	109 (26.7)	27 (34.2)	1.06	0.808	60 (55.0)	1.04	0.768
**Occupational status** ^**2**^								
Unemployed	97 (39.6)	203 (48.1)	39 (40.2)	ref.	-	100 (49.3)	ref.	-
Informal job	62 (25.3)	134 (31.8)	23 (37.1)	0.89	0.590	66 (49.3)	1.00	0.529
Employed	86 (35.1)	85 (20.1)	24 (27.9)	0.76	0.200	45 (52.9)	1.09	0.465
**Marital status** ^**2**^								
Married	74 (30.5)	137 (35.7)	29 (39.2)	ref.	-	66 (48.2)	ref.	-
Non-married	169 (69.5)	247 (64.3)	55 (32.5)	0.86	0.421	125 (50.6)	1.05	0.674

*Summary of variables in the line within gender by ^1^mean ± standard deviation or ^**2**^frequency (%).

**Summary of attempted suicide (*yes*) within rows and PR controlled by age and ethnicity.

Similar analyses were performed for all social and family-related variables (S1 Table), and all psychiatric, clinical and drug-related variables (S2 Table). For men, 18 variables had statistically significant prevalence ratios, and another set of 18 variables was detected in the women’s data. Eleven were significant in common to both genders, including hallucinations not under the effect of drug or abstinence, alcohol withdrawal symptoms, childhood physical abuse, drug withdrawal symptoms, being a victim of a violent crime, depressive disorder, childhood emotional abuse, and psychiatric hospitalization not related to alcohol or drug use, among a few others. The three highest PR out of the seven variables significant for men only were for physical abuse (PR = 2.14, p = .009), childhood physical neglect (PR = 1.74, p = .001), and having a close relationship with other drug dependent people (PR = 1.73, p = .010). Similarly, the highest PR for women were for difficulty controlling aggression (PR = 1.50, p = .001), childhood sexual abuse (PR = 1.36, p = .003), and chronic respiratory disease (PR = 1.30, p = .006).

**Fig 1:**
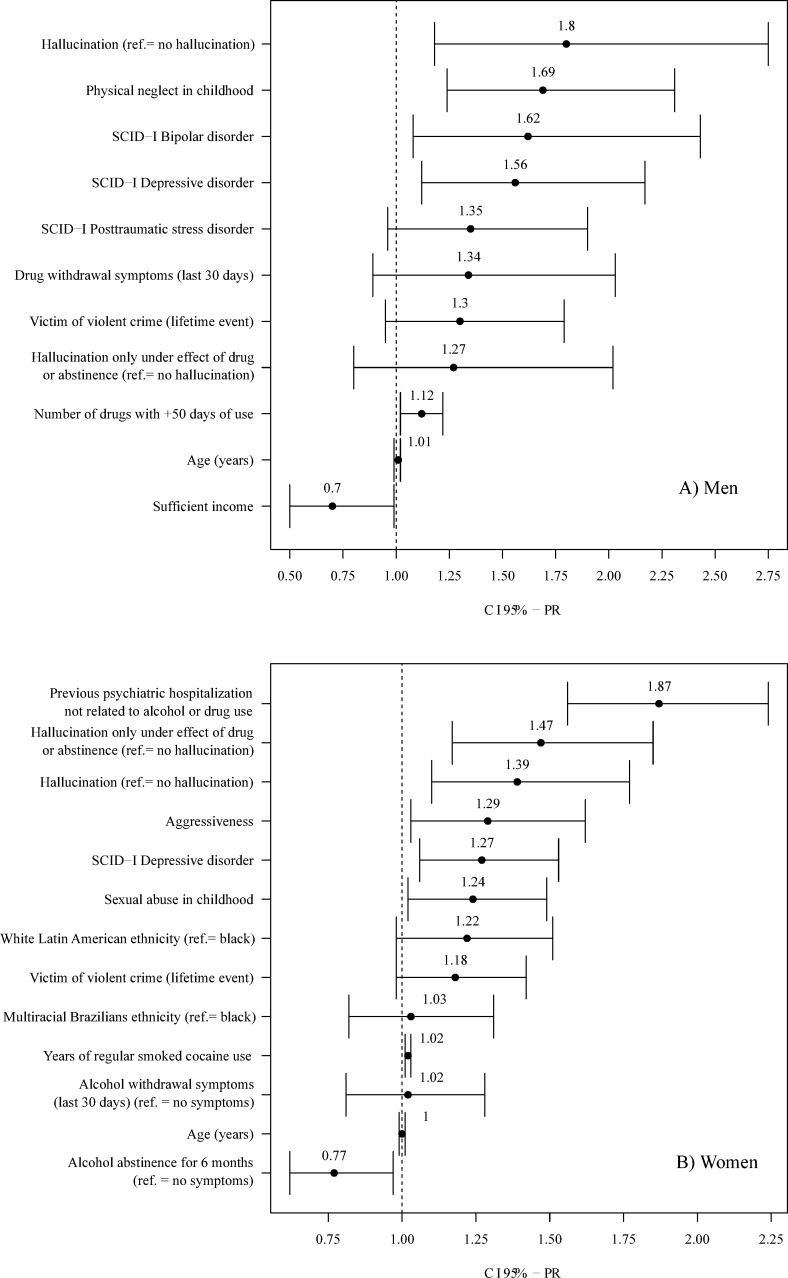
**Prevalence ratios and 95% confidence intervals of lifetime suicide attempt for the variables in the multiple poisson regression models among men (A; n = 246) and women (B; n = 402) crack-cocaine inpatient users.** The remaining variables in the last step of the backward elimination are shown. Variables with no indication of reference category (ref.) are binary (*yes/no*), and their reference category is *no*.

Fig 1 presents the final multiple GLMs, applying backward elimination to the respective 18 independent variables for each gender. Because of some missing data, the men's PRs were calculated with n = 246, where 6 out of 11 variables were statistically significant. For women, with n = 402, 8 out of 13 variables were statistically significant. The main results of this statistical approach include diagnosis of depression (PR_M_ = 1.56, p = .008; PR_W_ = 1.27, p = .011) and report of hallucinations throughout life (PR_M_ = 1.80, p = .006; PR_W_ = 1.39, p = .006), both of which remained significant factors associated with attempted suicide in men and women. The specific risk factor for each gender can be observed in the figure.

### Predictive approach

**Fig 2:**
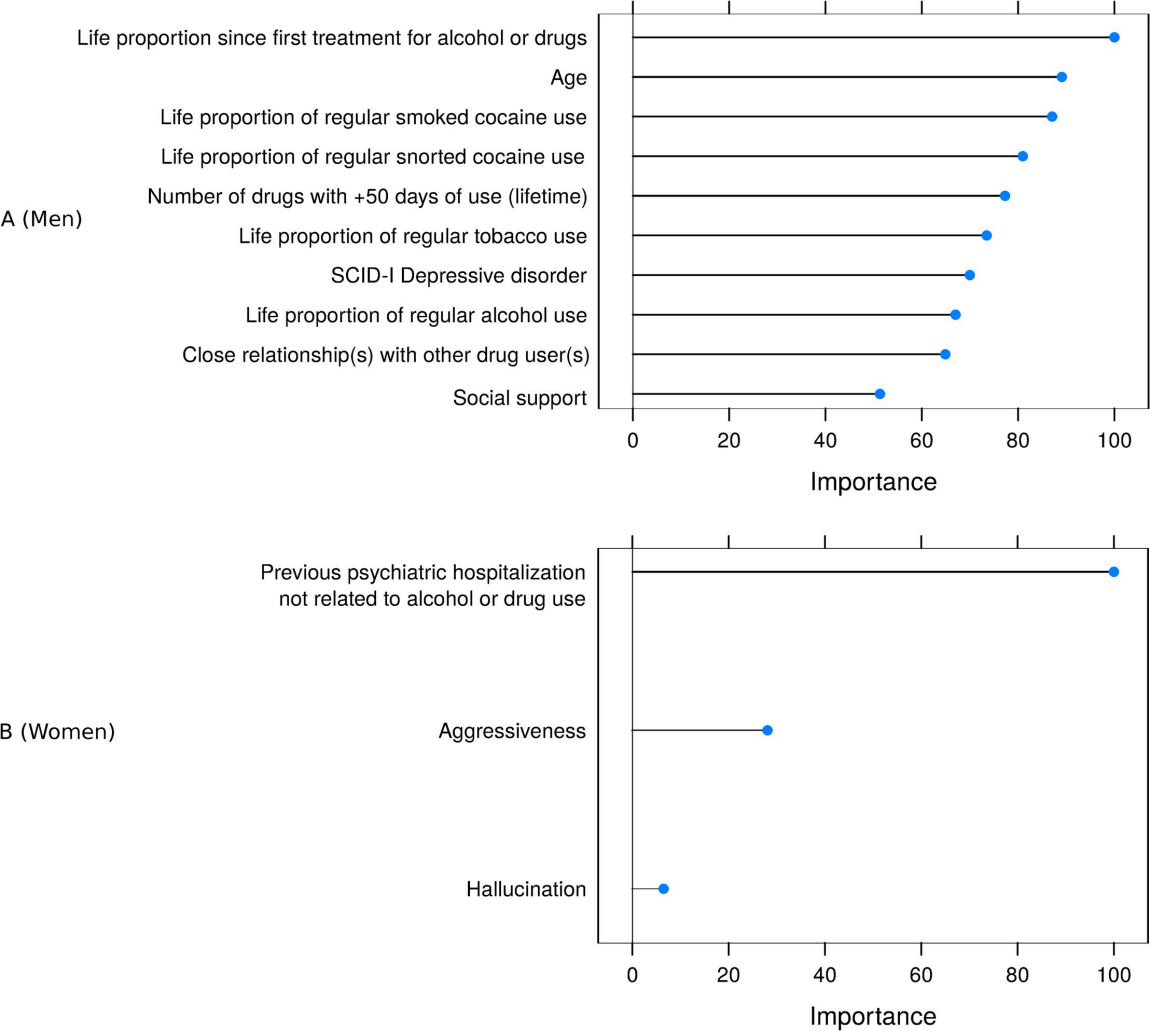
**ROC curves for the predictive models of attempted suicide plotted on the test data for each Random Forest learned model—men (A; n = 50) and women (B; n = 90).** The dot indicates the sensibility and specificity at whitch the distance between the curve and the transversal line is the largest.

For the machine learning approach, the RFE algorithm reduced the set of training features (predictors) from 57 to 27 for men and to only three for women. Fig 2 presents the ROC curves on the test data of the Random Forest fitted models for (A) men and (B) women. The AUCs were better than random in both cases (AUC_M_ = 0.680 and AUC_W_ = 0.734).

Table 2 presents the performance of the Random Forest models with the unseen test dataset. For women, some of the measures returned the same value, of 0.714, because the confusion matrix was symmetrical.

**Table 2:** Evaluation of the Random Forest models.

Gender	AUC	Sens.	Spec.	PPV	NPV	Bal. Acc.	NIR	H0: Acc. > NIR p-value
Men	0.680	0.823	0.500	0.467	0.842	0.662	0.653	0.765
Women	0.734	0.714	0.714	0.714	0.714	0.714	0.500	<0.001

AUC = Area under the receiver operating characteristic curve.

Sens. = Sensitivity. Spec. = Specificity. Bal. Acc. = Balanced Accuracy.

PPV = Positive Predictive Value. NPP = Negative Predictive Value.

NIR = No Information Rate.

According to the statistical test, the accuracy of the women’s model was significantly higher than NIR. Additionally, the balance between the evaluation metrics is further evidence toward good model performance. However, the accuracy of the men’s model was not significantly higher than NIR, which would imply that it does not predict attempted suicide better than using only the majority outcome category. It is advisable to interpret this result with caution, because the test does not consider all the statistics necessary to fully evaluate the model in order to ultimately conclude that it is not useful. Note that if all test instances were predicted as *no attempted suicide*, according to NIR, then sensitivity would be zero. The metrics for the category of interest should therefore be evaluated (Sens._M_ = 0.823 and PPV_M_ = 0.467).

**Fig 3:**
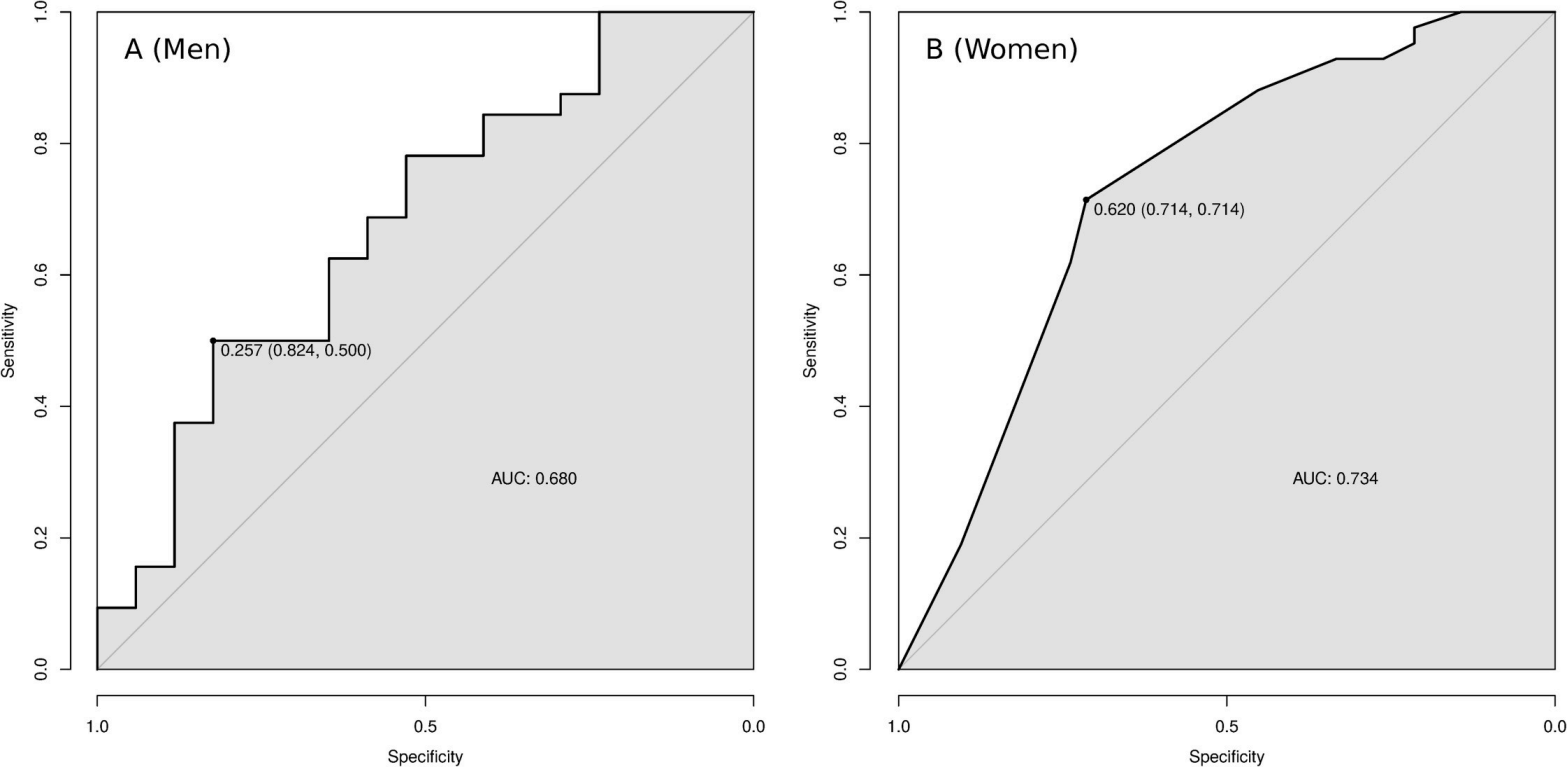
Variables with greater importance for the prediction of attempted suicide by the Random Forest algorithm according to gender. The top ten variables out of the 27 for men (A) and the three variables for women (B) are shown.

Fig 3 shows the ten most important variables for the men’s model and the three variables in the women’s model. In this approach, variables concerning an amount of time over lifetime, such as years of regular substance use, were divided by age to create the features of life proportion of regular use, detailed in the supplementary material S1 Text.

**Fig 4:**
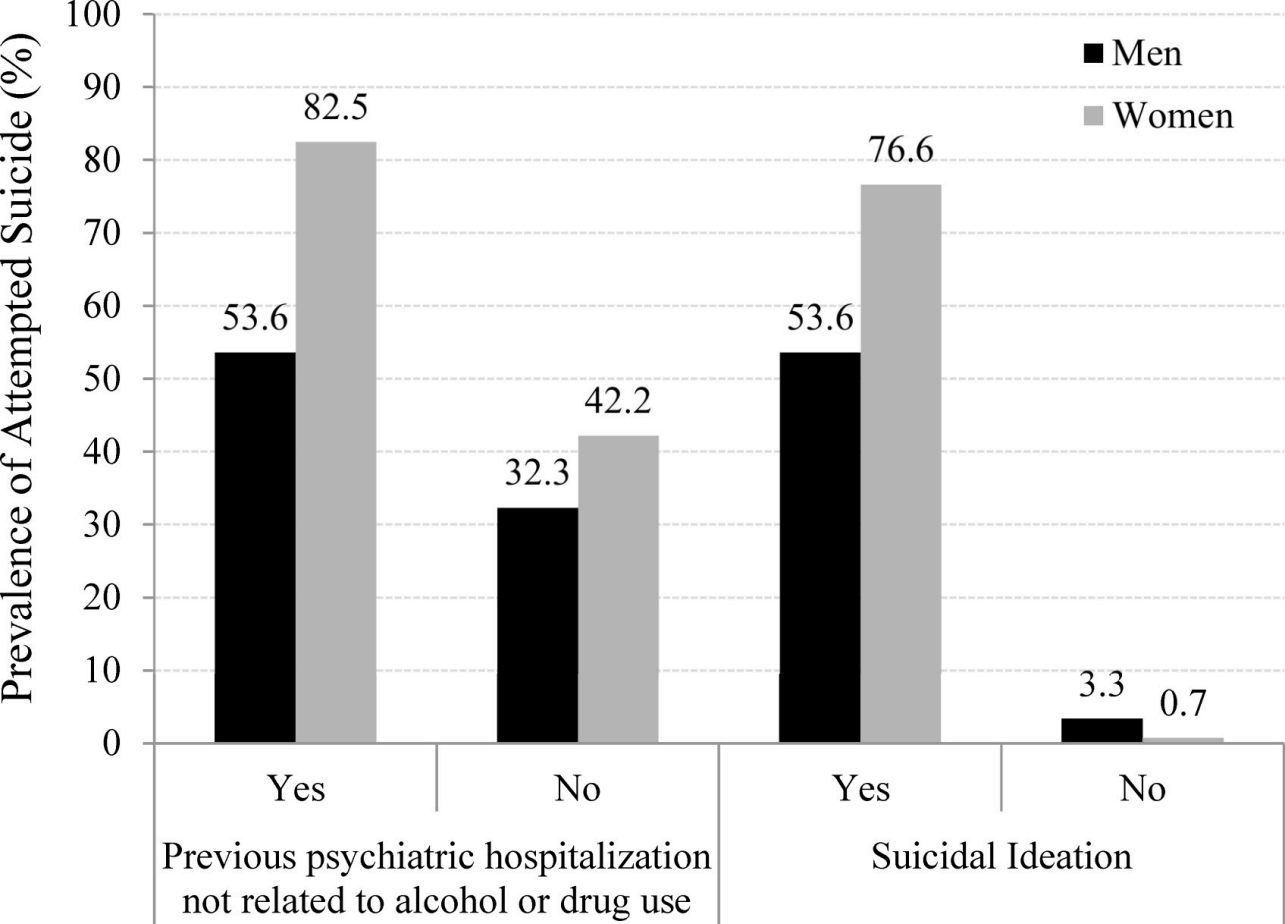
Prevalence of attempted suicide among men and women within the categories of suicidal ideation and previous psychiatric hospitalization (not related to alcohol or drug use).

We tested the inclusion of suicidal ideation as a predictor, with the result that all predictive statistics improved drastically. To illustrate the difference between the use of suicidal ideation and, for instance, previous psychiatric hospitalization not related to alcohol or drugs, Fig 4 shows the prevalence of attempted suicide between the combined categories of gender and those variables. Overall, the prevalence of attempted suicide was greater for those who presented a previous psychiatric hospitalization (not related to alcohol or drug) than for those who did not, and the difference was greater for women than for men (PR_M_ = 1.55, p = 0,031; PR_W_ = 1.91, p<0.001). However, the prevalence of attempted suicide for those who presented suicidal ideation was very much higher than for those who did not (PR_M_ = 15.8; PR_W_ = 112.6, both p<0.001), almost restricting the possible occurrence of attempted suicide to the presence of suicidal ideation. Both scenarios were expected, but in the case of the second, we conclude that it maintains a very proximal relationship with suicide, overlapping important distal factors and overfitting prediction without providing new knowledge. Therefore, it was included in neither approach.

## Discussion

To the best of our knowledge, this is the first study that conducts in-depth analyses of associated factors and prediction of attempted suicide in an inpatient sample of men and women with cocaine use disorder. The main findings of this study provide evidence to support the conclusion that men and women have different factors associated with attempted suicide, endorsing our decision to stratify by gender, and showing that complementary analytical approaches can provide additional insights and improve hypothesis generation. In women, our findings were more promising, with both analytical approaches showing agreement between risk factors associated with attempted suicide. In men the results were less consistent, where some variables were associated with the outcome in the descriptive model, although the predictive approach showed low accuracy.

Descriptive, explanatory, and predictive models are similar in practice but serve different analytical goals [49]. We presented factors associated with attempted suicide through a descriptive model approach that estimates prevalence ratios, and complementarily, a predictive model approach that explores those factors as predictors in a machine learning algorithm. The use of different approaches can help us to better understand the relationship structure between predictors and outcome. Although GLM can be used to explore such relationships using full factorial models, this strategy often suffers from poor statistical power when the sample size is not planned. As a result, it is very common to investigate linear relationships with main effect coefficients. In contrast, the Random Forest algorithm is a nonparametric method that relies on bootstrapping strategy to explore nonlinear relationships. Its weakness lies in the possibility of random results when applied to small samples, with which GLM often performs well. In this sense, using both approaches add robustness for confirmatory aspect and hypothesis generation. More than that, for some complex clinical questions, such as suicidal behavior, a linear thinking as conceptualized by the risk factors era with descriptive analyses may not solve them. Moreover, the accuracy of all the input data to predict the outcome is as important as the estimation of the impact of a specific predictor.

The descriptive analytical approach indicated that attempted suicide by individuals with cocaine use disorder is associated with some common factors among men and women. Among these factors, we highlight the presence of certain psychiatric symptoms and disorders (e.g. depression), abuse of psychoactive substances, psychiatric hospitalizations, and childhood maltreatment.

Of these associated factors, childhood trauma appears to play a critical role. A large cohort study showed that childhood trauma increased by 2 to 5 times the risk of suicide attempt across the lifespan [50]. More than that, several studies have demonstrated that childhood trauma is highly associated with suicide attempts in several psychiatric disorders, such as bipolar disorder, depression, schizophrenia [51], and drug addiction [1,52,53]. A recent meta-analysis of longitudinal studies reported higher rates of childhood maltreatment in individuals with SUD compared to the general population [54]. In our study, physical and sexual abuses during childhood were associated with suicide attempt in both genders. An association between physical, sexual and emotional abuse and attempted suicide was also seen in different populations, such as subjects with bipolar disorders [55], prisoners [56], and women in custody [57], and was also related to increased risk of psychiatric disorder across lifespan [58].

Other factors we identified in association with attempted suicide among men and women were major depressive disorders and hallucinations. Mood disorders are commonly accompanied by SUD, leading to worse course of illness and several functional and cognitive impairments [59,60]. Studies suggest that mood disorders and SUD share common genetic predispositions and environmental triggers that may be related to the chronic trajectory and severity of disorders [61]. It is well established that psychopathology is a precipitating risk factor for suicide since 90% of individuals who died from suicide had some type of psychiatric disorder [1]. Furthermore, it is plausible that hallucinations and psychotic experiences may lead individuals with SUD to attempt suicide. A cross-sectional study, for example, demonstrated that auditory verbal hallucinations increased the odds of suicide attempt in adolescents with suicidal ideation regardless of gender [62,63].

By the other hand, there were also different associated factors with attempted suicide among men and women. In men, exclusive factors included bipolar disorder, insufficient income and number of drugs used for more than 50 days over lifetime. Evidence indicates that substance abuse expresses a higher severity of bipolar disorder and drug use behavior may be a mechanism of emotional regulation typical of this disorder [64,65]. Concurrently, other authors indicate that impulsivity or coping skills may mediate drug use [66–68]. Furthermore, our findings are corroborated by other studies which found evidence that subjects in precarious employment and with low incomes had a higher risk of suicide [69,70]. A recent systematic review found that worse economic status and unemployment has been associated with suicidal behavior [71]. Lack of economic resources seems to increase the burden of the preexisting clinical condition and it is common for users of a highly addictive drug to spend a significant part of their income on acquiring that drug [72–74]. This cycle most probably prolongs the burden related to economic vulnerability as long as drug use addictive behavior remains.

Looking at the Random Forest model of male crack-cocaine users, the most important variables are related to drug addiction severity and poor social-family support. In this context, drug severity related factors could act as moderators of the relationship between other variables and attempted suicide, as well as themselves being predictors. This fact also reflects a more complex model given the homogeneous importance assigned to many variables. The most important predictor for men was life proportion since the first treatment for alcohol or drugs, which is consistent with previous studies [75]. In a national survey in the United States, the number of substances had greater importance for predicting suicide attempts than the type of substances used [76].

For women, exclusive variables in the descriptive approach included difficulty in controlling aggression, presence of abstinence symptoms, and regular years of snorted cocaine use. Interestingly, a study with a community sample of adults revealed that high levels of withdrawal symptoms increased the risk of suicide in women, but not in men [77]. Alcohol-withdrawal symptoms could be considered as a stressor factor since drug withdrawal leads to emotional and physical aversive states. These long-term effects might work as unpredictable and uncontrollable stressors, leading to a process of stress sensitization and enhancement of the endogenous aversive state due to drug withdrawal [78,79]. Thus, these subjects may not have good coping skills for facing the severity of withdrawal symptoms, leading to suicide attempts [77,80].

Our findings also associated the number of years of regular smoked-cocaine use and previous psychiatric hospitalization not related to alcohol or drug use with suicide in women. Both variables are understood as hallmarks of illness chronicity in psychiatric conditions [81]. In the neuroprogression model, the number of psychiatric episodes, number of hospitalizations, substance use, and childhood trauma contribute to impaired response to treatment and cognitive and psychosocial functioning and to higher rates of suicide attempt [82,83]. We also found that aggression was significantly associated with attempted suicide in women. This factor could have an overlap with suicidal behavior, interpersonal aggression, and self-harm as a spectral event, in which some subjects may engage in one of these different types of violent behavior [84,85]. Interestingly, it is suggested that aggression and impulsivity are diathesis factors in suicidal behavior [86,87].

Moreover, the women’s predictive model was much easier to interpret, had higher predictive power, and was closer to a parsimonious model, which involves the minimum possible number of parameters to be estimated while thoroughly explaining the behavior of the outcome. Here, previous psychiatric hospitalization not related to alcohol or drugs, aggression, and hallucinations were the most important variables for predicting attempted suicide, drawing attention to other psychiatric events in this subpopulation and corroborating descriptive results for this group.

Regarding treatment, both suicidal behavior and addiction involve several problematic aspects in the individual's life, one being aggravated by the other, so intensive psychotherapy is highly recommended in order to rearrange the lives of these people as a whole. As our sample has shown, this population has a high prevalence of attempted suicide and is already at risk of suicide, so approaches focused on suicide prevention are needed early on. A randomized controlled trial of individuals who attempted suicide showed that cognitive therapy was effective in preventing suicide reattempt and depression [88]. However, there is no focus on gender-related psychiatric differences in several studies about suicide despite having gender distinctness. Hence, it urges to develop tailored strategies focused on suicide risk factors according to gender as implementing specific mental health policies among men and women, eliminating health care access barriers, and restricting access to lethal means [89]. Following our specific findings on gender differences, psychosocial treatments should be targeted coping strategies and social skills due to the less social support common in men. While in women, it should be tailored on preventing psychiatric comorbidities and encouraging anger management. In relation to pharmacological treatment, lithium was effective to prevent suicide in subjects with mood disorder as shown in a meta-analysis of randomized clinical trials [90]. But for its administration in cocaine addicts, further studies with this population are needed to support this treatment alternative.

## Limitations

Data were based on retrospective self-report of attempted suicide, and subjects may underreport data and have biased recall, especially in a SUD sample. In the predictive approach, the model for men did not achieve satisfactory overall classification accuracy. This result could be caused by many factors, such as a weak explanation of attempted suicide by the set of variables used or, and more plausible, due to the small sample size in terms of the needs for effective fitting models based on supervised machine learning. These circumstances reflect insufficient information for high precision. However, this is nevertheless one of the largest clinical samples of cocaine use disorder inpatients. Also, suicide attempts are more prevalent in women than men, which could be explained by the fact that men tend to use more lethal methods, leading to greater suicide mortality than women [68]. The prevalence difference translates into a class imbalance problem [91], which, although it was addressed in model training, still leaves the test data unbalanced and the overall classification accuracy less meaningful for evaluating the model than class-specific measures, such as sensitivity and PPV. We suggest that further studies should focus on predictive models of both suicide attempt and death in order to achieve more robust findings. Furthermore, the study design does not allow for causal conclusions between predictors and suicide but explores the relationship between the variables. These limitations are due to the use of secondary databases, because the factors associated with attempted suicide captured by the predictive models were limited to the variables available in the data collection instruments. A measure of impulsivity, for instance, is a highly desirable variable for predictive suicide studies. It could help to explain suicide attempts that occurred without suicidal ideation. Factors that aggravate vulnerabilities and stress, such as quality of life and sexual orientation, among many others, would also be desirable.

## Conclusions

Our findings indicate that attempted suicide is associated with depression and hallucinations in both genders, as well as previous hospitalization for mental issues—related to psychiatry for women and indirectly related to substance abuse for men. This scenario suggests that the severity of drug use may be a moderator between predictors and suicide among men. Our study also corroborates the influence of childhood trauma on suicide attempts, with different types of trauma being related to risk factors for both men and women. It is noteworthy that both of our analytical approaches showed excellent consistency with respect to the variables associated with suicide among women.

We hypothesize that highly accurate predictive models would support important clinical decisions such as suicide prediction, selection of treatment options, and prognosis orientations. The present study advances the field of machine learning in mental disorders and presents a model for prediction of attempted suicide in patients with cocaine use disorder. Future longitudinal studies should replicate these findings using larger samples from multiple centers and pursue a data fusion approach, combining clinical data with other biological measures, such as genetics, to build more accurate tools to predict suicide attempts in subjects with cocaine use disorder.

## Supporting information

S1 Text Detailed information on the methods for the machine learning approach. (DOCX)

S1 Fig Missing data pattern of the selected sample. Each line represents a variable, each column a participant, and the red dots mark the missing data along with the histogram of their frequency. Only 4 out of 57 variables have more than 3% of data missing.
(EPS)

S2 Fig Comparison of the distributions of original (blue) and inputted (red) training data by MICE method for (A) men and (B) women.
(EPS)

S1 Table Prevalence ratios of attempted suicide for social and family-related variables, stratified by gender, controlled by age and ethnicity. (DOCX)

S2 Table Prevalence ratios of attempted suicide for psychiatric, clinical and drug-related variables, stratified by gender, controlled by age and ethnicity. (DOCX)
