# Supplementary material for: Prediction of attempted suicide in men and women with crack-cocaine use disorder in Brazil
Source: PLoS One. 2020 May 4;15(5):e0232242. doi: 10.1371/journal.pone.0232242 (PMC7197800; doi:10.1371/journal.pone.0232242)
Supplement: S1 Text — (DOCX) [file pone.0232242.s001.docx]

**Text S1.** Detailed information on the methods for the machine learning approach.

**Methods**

***Participants and assessments***

Data were collected using three instruments: the sixth version of the Addiction Severity Index (ASI-6); the Structured Clinical Interview for DSM-IV axis I disorders (SCID-I); and the Childhood Trauma Questionnaire (CTQ).

ASI-6 was developed by Thomas McLellan [25] and validated in Brazil by Kessler et al. [26]. It is a structured interview lasting approximately 45 minutes and addresses the impact of substance use in the last 30 days, last six months, and over lifetime. The instrument comprises 243 questions regarding seven working areas: Medical, Employment and Support, Alcohol use, Drug use, Legal aspects, and Socio-familiar and Psychiatric aspects. The instrument also contains a section of sociodemographic information.

The Clinical Interview for Axis I Disorders of the Diagnostic and Statistical Manual of Mental Disorders IV (SCID-I) was adapted for Brazil by Del-Ben et al. [27]. This instrument consists of a semi-structured interview lasting approximately 60 minutes and provides presence or absence of diagnoses of current and past psychiatric disorders of Axis I according to criteria of the fourth version of the Diagnostic and Statistical Manual of Mental Disorders. It is divided into six modules: mood episodes, psychotic episodes, psychotic disorders, mood disorders, substance use disorders, anxiety, and other disorders.

The Childhood Trauma Questionnaire (CTQ) was developed by Bernstein et al. [28] and validated in Brazil by Grassi-Oliveira et al. [29]. This instrument can be self-applied in 8 minutes and graduates the frequency of 28 assertions related to childhood situations on a five-point Likert scale, investigating five traumatic components: childhood physical abuse (PA), emotional abuse (EA), sexual abuse (SA), physical neglect (FN), and emotional neglect (EN).

***Preprocessing and data transformation***

The new variables created based on changes and combinations of the original ones are:

1. Number of chronic diseases. ASI6’s second section consists of medical issues. Questions M3-M14 explore by self-report high pressure (M3), diabetes (M4), heart disease (M5), stroke or ischemia (M6), epilepsy or convulsions (M7), cancer (M8), HIV or AIDS (M9), tuberculosis (M10), hepatitis (M11), cirrhosis or other chronic liver disease (M12), chronic kidney disease (M13) and chronic respiratory problem (M14) with the question “Have you ever been told by a doctor or healthcare provider that you had any of the following physical or medical conditions”? This variable was created using the sum of those, except for M10, M11, and M14, that were used individually; and also M9, that was replaced by anti-HIV test. So, this is a discrete variable ranging from 0 to 8.
2. Life proportion since the first treatment for alcohol or drugs. In the ASI6’s section about Alcohol and Drugs, D03 question asks, “How old were you the first time you entered alcohol or drug abuse treatment”? To create this variable, we divided this answer by age, so it is a continuous variable ranging from 0 to 1.
3. Life proportion of regular use of alcohol, marijuana, snorted and smoked cocaine, and tobacco. Here, we have five variables, calculated similarly to the previous ones. Questions D08 and D09 ask, “How many years in your life have you drank alcohol on a regular basis, 3+ days per week?” and “How many years in your life have you drank at least (5-men, 4-women) drinks per day on a regular basis, 3+ days per week?”, respectively. We used the maximum of those two divided by age to create the life proportion of regular use of alcohol. Variables D25B, D27B, and D28B ask the same as D08 but related to marijuana, snorted cocaine, and smoked cocaine instead of alcohol, respectively. D57 refers to years of daily use of tobacco. All of those divided by age create the life proportion of regular use of the respective substance, also continuous variables ranging from 0 to 1.
4. Number of psychoactive substances used for more than 50 days (lifetime). Also in the Alcohol and Drugs section of ASI6, the questions D25C through D33C ask “Have you used [substance] on 50 or more days in your life?” for the following substances: marijuana (D25C), sedatives (D26C), snorted cocaine (D27C), smoked cocaine (D28C), stimulates (D29C), hallucinogens (D30C), heroin (D31C), other opioids (D32C) and inhalants (D33C). The question D10 asks, “Have you drank at least (5-men, 4-women) drinks in a day in 50 or more days in your life”? Finally, we dichotomize D57 (0 = no tobacco; 1 through higher = yes) and sum all of those in this new variable, creating a discrete variable ranging from 1 to 11.
5. Social support network. Another section of ASI6 has questions about Family and Social. Questions F9A, F9B, and F9C ask, “If you need help, can you count on: (A/B/C)?”, where person A is partner, B is adult relatives and C is close friends (one binary answer for each). Also, question F12 asks, “Aside from your partner, other adult relatives and close friends, are there any people you keep in touch with that you can count on if you really need help”? The sum of these four answers creates this variable, which is a discrete variable ranging from 0 to 4.
6. Close relationship(s) with other drug user(s). Similarly to the previous variable, the questions F8A, F8B, and F8C ask, “Do/does your A/B/C have a current problem with alcohol or use drugs?”, where person A is partner, B is adult relatives, and C is close friends (one binary answer for each). The sum of these three answers creates this variable, which is a discrete variable ranging from 0 to 3.
7. Difficulty in controlling aggressiveness. ASI6’s P13A and P14A questions ask, “Have you ever (since 18) had difficulty controlling your temper, or urges to hit or harm someone?” and “Have you ever (since 18) pushed, hit, thrown things at, or used weapons against someone?”, respectively. This is a binary variable (0=no; 1=yes) created by the answer “yes” for at least one of the questions.
8. Dichotomized CTQ scores. Each trauma is composed of a sum of 5 Likert statements about the respective trauma construct. There is a specific threshold for each trauma being considered present, as follows: PN>9, AP>9, EN>14, EA>12, and SA>7.
9. Physical abuse in childhood. According to CTQ’s application instructions, the questions must be answered concerning life before 16 years to be considered in childhood. Following this threshold, we combined ASI6’s questions F23 and F29, which ask “Have you ever been physically assaulted/abused by someone you knew?” and “Have you ever been the victim of a violent crime like being mugged, assaulted?” with their subsequent questions, F24 and F30. They ask, “How old were you when this first happened”? We merge these data considering under 16 years old with CTQ’s dichotomized physical abuse to create this new binary variable (0=no; 1=yes).
10. Sexual abuse in childhood. This variable was created similarly to the previous one, combining CTQ’s dichotomized sexual abuse variable and ASI6’s F26 and F27 questions, which ask “Have you ever been sexually assaulted/abused by someone you knew?” and age of the first occurrence, respectively. This combination results in a binary variable.
11. Physical abuse in a lifetime. This variable is binary and was created by the answers “yes” for at least one of ASI6’s F24 and F29 questions or CTQ’s dichotomized childhood physical abuse.
12. Sexual abuse in a lifetime. Similarly to the previous, this variable uses only ASI’s F26 question together with CTQ’s dichotomized childhood sexual abuse.
13. Idealization of upbringing. A numerical variable that sums the answers of the CTQ statements 10, 16 and 22, which are not used to compute any trauma score and is used to assess issues related to social desirability or a tendency to deny negative experiences during childhood.
14. SICD-I diagnoses. They were grouped and dichotomized as psychotic, bipolar, depression, obsessive-compulsive, post-traumatic stress, alcohol use, eating and anxiety disorders.

***Data mining and statistical analysis***

*Machine learning approach*

Figure S2 presents the density plots of numeric variables comparing the original (blue) and the imputed (red) distribution in training data for (A) men and (B) women. This algorithm creates multiple replacement values for multivariate missing data based on Fully Conditional Specification, where a separate model imputes each incomplete variable. After that, we compute the average for numeric variables, median for ordinal categorical, and mode for nominal ones. These calculated values were used to impute the test data. That is, for each variable, all test instances were imputed with the same value. The chosen imputation strategy prevents any test data from being used to learn the classification model. Furthermore, it considers each test instance alone without using any statistics on the test set.
