# Supplementary material for: Prediction of attempted suicide in men and women with crack-cocaine use disorder in Brazil
Source: PLoS One. 2020 May 4;15(5):e0232242. doi: 10.1371/journal.pone.0232242 (PMC7197800; doi:10.1371/journal.pone.0232242)
Supplement: S1 Table — (DOCX) [file pone.0232242.s004.docx]

**Table S1.** Prevalence ratios of attempted suicide for social and family-related variables, stratified by gender, controlled by age and ethnicity.

|  | **Gender*** | |  | **Attempted Suicide**** | | | | | | |
| --- | --- | --- | --- | --- | --- | --- | --- | --- | --- | --- |
|  | **Men** | **Women** |  | **Men** | **PR** | **p-value** |  | **Women** | **PR** | **p-value** |
| **Sufficient income** | 119 (48.2) | 159 (38.5) |  | 31 (26.1) | 0.61 | 0.007 |  | 83 (52.2) | 1.07 | 0.532 |
| **Have children** | 165 (66.8) | 364 (87.3) |  | 59 (35.8) | 0.95 | 0.792 |  | 176 (48.4) | 0.79 | 0.064 |
| **Have an authentic/genuine friendship** | 141 (57.8) | 200 (47.6) |  | 50 (35.5) | 1.04 | 0.812 |  | 103 (51.5) | 1.08 | 0.420 |
| **Social Support** |  |  |  |  |  |  |  |  |  |  |
| ..Have no support from partner, close friends or adult relatives | 7 (2.8) | 26 (6.2) |  | 2 (28.6) | ref. | - |  | 11 (42.3) | ref. | - |
| ..One person supports him/her | 66 (26.7) | 121 (28.7) |  | 25 (37.9) | 1.45 | 0.535 |  | 55 (45.5) | 1.07 | 0.786 |
| ..Two people support him/her | 93 (37.7) | 156 (37) |  | 28 (30.1) | 1.13 | 0.838 |  | 83 (53.2) | 1.28 | 0.303 |
| ..Three people support him/her | 59 (23.9) | 90 (21.3) |  | 24 (40.7) | 1.58 | 0.446 |  | 49 (54.4) | 1.29 | 0.312 |
| ..Four people support him/her | 22 (8.9) | 29 (6.9) |  | 7 (31.8) | 1.15 | 0.831 |  | 13 (44.8) | 1.09 | 0.779 |
| **Close relationship(s) with other drug user(s)** |  |  |  |  |  |  |  |  |  |  |
| ..Partner, friends and adult relatives are not drug dependent | 115 (46.6) | 160 (37.9) |  | 31 (27.2) | ref. | - |  | 72 (45.9) | ref. | - |
| ..One of them is drug dependent | 82 (33.2) | 180 (42.7) |  | 30 (36.6) | 1.36 | 0.142 |  | 91 (50.6) | 1.10 | 0.394 |
| ..Two or three of them are drug dependent | 50 (20.2) | 82 (19.4) |  | 24 (48.0) | 1.73 | 0.010 |  | 45 (54.9) | 1.18 | 0.211 |
| **Homeless (lifetime event)** | 113 (48.9) | 185 (54.3) |  | 46 (40.7) | 1.39 | 0.066 |  | 94 (50.8) | 1.00 | 0.937 |
| **Arrested (lifetime event)** | 97 (39.6) | 93 (22.1) |  | 32 (33.0) | 0.94 | 0.713 |  | 41 (44.1) | 0.85 | 0.202 |
| **Difficulty in talking about your feelings** | 133 (54.1) | 277 (67.1) |  | 52 (39.1) | 1.31 | 0.135 |  | 141 (50.9) | 1.17 | 0.248 |
| **Difficulty in enjoying your leisure time** | 167 (68.4) | 257 (62.2) |  | 65 (38.9) | 1.40 | 0.115 |  | 132 (51.4) | 1.08 | 0.460 |
| **Difficulty in controlling aggressiveness** | 151 (61.6) | 280 (66.7) |  | 56 (37.1) | 1.19 | 0.354 |  | 156 (55.7) | 1.50 | 0.001 |
| **Victim of violent crime (lifetime event)** | 100 (41.0) | 135 (32.3) |  | 46 (46.0) | 1.83 | <0.001 |  | 80 (59.3) | 1.28 | 0.010 |
| **Saw someone being killed** | 195 (78.9) | 248 (59.2) |  | 73 (37.4) | 1.59 | 0.076 |  | 125 (50.4) | 1.02 | 0.822 |
| **Childhood physical neglect** | 84 (34.0) | 148 (35.1) |  | 41 (48.8) | 1.74 | 0.001 |  | 76 (51.4) | 1.03 | 0.752 |
| **Childhood emotional neglect** | 51 (20.6) | 131 (31.0) |  | 24 (47.1) | 1.47 | 0.051 |  | 70 (53.4) | 1.08 | 0.497 |
| **Childhood emotional abuse** | 103 (41.7) | 183 (43.4) |  | 46 (44.7) | 1.61 | 0.006 |  | 102 (55.7) | 1.21 | 0.048 |
| **Physical abuse in life** | 187 (75.7) | 349 (82.7) |  | 74 (39.6) | 2.14 | 0.009 |  | 183 (52.4) | 1.35 | 0.062 |
| **Childhood physical abuse** | 156 (63.2) | 255 (60.4) |  | 67 (42.9) | 2.17 | 0.001 |  | 141 (55.3) | 1.31 | 0.012 |
| **Sexual abuse in life** | 59 (23.9) | 239 (56.6) |  | 27 (45.8) | 1.41 | 0.052 |  | 131 (54.8) | 1.24 | 0.041 |
| **Childhood sexual abuse** | 59 (23.9) | 209 (49.5) |  | 27 (45.8) | 1.41 | 0.052 |  | 121 (57.9) | 1.36 | 0.003 |
| *Summary of variables in the line within gender by frequency (%). | | | | | | | | | | |
| **Summary of attempted suicide (*yes*) within rows and PR controlled by age and ethnicity. Ref.= reference category (*no* for binary variables). | | | | | | | | | | |
