# Supplementary material for: Prediction of attempted suicide in men and women with crack-cocaine use disorder in Brazil
Source: PLoS One. 2020 May 4;15(5):e0232242. doi: 10.1371/journal.pone.0232242 (PMC7197800; doi:10.1371/journal.pone.0232242)
Supplement: S2 Table — (DOCX) [file pone.0232242.s005.docx]

**Table S2.** Prevalence ratios of attempted suicide for psychiatric, clinical and drug-related variables, stratified by gender, controlled by age and ethnicity.

|  | **Gender*** | |  | **Attempted Suicide**** | | | | | | |
| --- | --- | --- | --- | --- | --- | --- | --- | --- | --- | --- |
|  | **Men** | **Women** |  | **Men** | **PR** | **p-value** |  | **Women** | **PR** | **p-value** |
| **HIV ²** | 23 (9.3) | 78 (18.5) |  | 7 (30.4) | 0.77 | 0.428 |  | 39 (50.0) | 0.99 | 0.955 |
| **Tuberculosis ²** | 17 (6.9) | 25 (5.9) |  | 6 (35.3) | 0.93 | 0.837 |  | 12 (48.0) | 0.95 | 0.801 |
| **Hepatitis ²** | 31 (12.6) | 45 (10.8) |  | 15 (48.4) | 1.30 | 0.230 |  | 24 (53.3) | 1.08 | 0.609 |
| **Chronic respiratory disease ²** | 41 (16.6) | 155 (36.8) |  | 18 (43.9) | 1.34 | 0.138 |  | 91 (58.7) | 1.30 | 0.006 |
| **Age at first alcohol use ¹** | 14.3 ± 2.8 | 15.4 ± 4.5 |  | - | 0.96 | 0.216 |  | - | 1.01 | 0.336 |
| **Years of regular alcohol use ³** | 2 [0-11] | 0 [0-4] |  | - | 1.02 | 0.042 |  | - | 1.01 | 0.204 |
| **Alcohol withdrawal symptoms (last 30 days) ²** |  |  |  |  |  |  |  |  |  |  |
| .. No symptoms | 36 (14.8) | 62 (14.9) |  | 33 (29.5) | ref. | - |  | 106 (52.5) | ref. | - |
| .. Abstinent for 6 months | 112 (45.9) | 202 (48.7) |  | 28 (29.2) | 0.91 | 0.657 |  | 61 (40.4) | 0.76 | 0.024 |
| ..Yes | 96 (39.3) | 151 (36.4) |  | 24 (66.7) | 2.15 | <0.001 |  | 39 (62.9) | 1.21 | 0.101 |
| **Alcohol craving (last 30 days) ²** | 56 (23.0) | 87 (20.6) |  | 26 (46.4) | 1.38 | 0.090 |  | 53 (60.9) | 1.28 | 0.017 |
| **Age at first cannabis use ¹** | 15.2 ± 4.4 | 15.4 ± 4.5 |  | - | 1.00 | 0.842 |  | - | 1.00 | 0.957 |
| **Years of regular cannabis use ³** | 7 [2-13.5] | 4 [0-12] |  | - | 1.00 | 0.681 |  | - | 1.00 | 0.783 |
| **Age at first snorted cocaine use ¹** | 18.1 ± 4.6 | 18.5 ± 5.9 |  | - | 0.99 | 0.862 |  | - | 1.00 | 0.849 |
| **Years of regular snorted cocaine use ³** | 5.5 [2-13] | 4 [0-10] |  | - | 1.01 | 0.420 |  | - | 1.02 | 0.026 |
| **Age at first smoked cocaine use ¹** | 24.6 ± 8.1 | 19.1 ± 7.2 |  | - | 1.00 | 0.953 |  | - | 1.00 | 0.656 |
| **Years of regular smoked cocaine use ³** | 6 [3-11] | 5 [2-10] |  | - | 1.00 | 0.899 |  | - | 0.99 | 0.334 |
| **Drug withdrawal symptoms (last 30 days) ²** | 146 (61.1) | 302 (72.2) |  | 63 (43.2) | 1.99 | 0.002 |  | 161 (53.3) | 1.31 | 0.032 |
| **Drug craving (last 30 days) ²** | 161 (66.5) | 301 (73.1) |  | 61 (37.9) | 1.24 | 0.274 |  | 161 (53.5) | 1.26 | 0.066 |
| **Age at first tobacco use ¹** | 14.2 ± 3.7 | 13.7 ± 4 |  | - | 1.01 | 0.639 |  | - | 0.99 | 0.622 |
| **Years of daily tobacco use ³** | 14 [4-22] | 15 [8-21] |  | - | 1.02 | 0.064 |  | - | 0.99 | 0.692 |
| **Number of psychoactive drugs used for more than 50 days in life ¹** | 4.5 ± 1.5 | 4.2 ± 1.3 |  | - | 1.22 | <0.001 |  | - | 1.09 | 0.019 |
| **Age at first treatment for alcohol and/or drugs ¹** | 28.1 ± 8.7 | 24.5 ± 8.3 |  | - | 0.99 | 0.655 |  | - | 1.00 | 0.940 |
| **Previous psychiatric hospitalization not related to alcohol or drug use ²** | 28 (11.4) | 80 (19.0) |  | 15 (53.6) | 1.55 | 0.031 |  | 66 (82.5) | 1.93 | <0.001 |
| **Hallucination (lifetime event) ²** |  |  |  |  |  |  |  |  |  |  |
| ..No | 116 (47.0) | 202 (48.6) |  | 24 (20.7) | ref. | - |  | 74 (36.6) | ref. | - |
| ..Yes | 54 (21.9) | 89 (21.4) |  | 29 (53.7) | 2.53 | <0.001 |  | 60 (67.4) | 1.85 | <0.001 |
| ..Only under effect of drug or abstinence | 77 (31.2) | 125 (30.0) |  | 33 (42.9) | 1.97 | 0.002 |  | 72 (57.6) | 1.56 | <0.001 |
| **Felt yourself incapacitated due to psychological or psychiatric issues (last 30 days) ²** | 86 (44.1) | 168 (45.0) |  | 36 (41.9) | 1.18 | 0.366 |  | 96 (57.1) | 1.12 | 0.257 |
| **Suicidal Ideation** | 155 (63.0) | 263 (64.8) |  | 83 (53.6) | 15.8 | <0.001 |  | 209 (76.6) | 112.6 | <0.001 |
| **SCID-I Psychotic disorder ²** | 40 (16.2) | 33 (7.8) |  | 18 (45.0) | 1.24 | 0.274 |  | 20 (60.6) | 1.22 | 0.182 |
| **SCID-I Depressive disorder ²** | 67 (27.1) | 131 (31.0) |  | 36 (53.7) | 1.83 | <0.001 |  | 78 (59.5) | 1.30 | 0.006 |
| **SCID-I Bipolar disorder ²** | 15 (6.1) | 42 (10.0) |  | 9 (60.0) | 1.69 | 0.020 |  | 26 (61.9) | 1.24 | 0.119 |
| **SCID-I Alcohol use disorder ²** | 159 (64.4) | 245 (58.1) |  | 64 (40.3) | 1.53 | 0.046 |  | 133 (54.3) | 1.24 | 0.044 |
| **SCID-I Eating disorder ²** | 7 (2.8) | 10 (2.4) |  | 1 (14.3) | 0.46 | 0.418 |  | 5 (50.0) | 0.97 | 0.932 |
| **SCID-I Anxiety disorder ²** | 121 (49.0) | 262 (62.1) |  | 48 (39.7) | 1.30 | 0.133 |  | 138 (52.7) | 1.15 | 0.176 |
| **SCID-I Post-traumatic stress disorder ²** | 26 (10.5) | 111 (26.3) |  | 16 (61.5) | 1.71 | 0.008 |  | 59 (53.2) | 1.10 | 0.389 |
| **SCID-I Obsessive-Compulsive disorder ²** | 16 (6.5) | 78 (18.5) |  | 9 (56.3) | 1.55 | 0.073 |  | 40 (51.3) | 1.02 | 0.887 |
| *Summary of variables in the line within gender by ¹mean ± standard deviation, ²frequency (%) or ³median [quartile1-quartile3]. | | | | | | | | | | |
| **Summary of attempted suicide (*yes*) within rows and PR controlled by age and ethnicity. Ref.= reference category (*no* for binary variables). | | | | | | | | | | |
